# Topology and control of self-assembled domain patterns in low-dimensional ferroelectrics

**DOI:** 10.1038/s41467-020-19519-w

**Published:** 2020-11-13

**Authors:** Y. Nahas, S. Prokhorenko, Q. Zhang, V. Govinden, N. Valanoor, L. Bellaiche

**Affiliations:** 1 Physics Department and Institute for Nanoscience and Engineering, University of Arkansas, Fayetteville, AR 72701 USA; 2 School of Materials Science and Engineering, University of New South Wales, Sydney, NSW 2052 Australia

**Keywords:** Two-dimensional materials, Ferroelectrics and multiferroics, Phase transitions and critical phenomena

## Abstract

Whilst often discussed as non-trivial phases of low-dimensional ferroelectrics, modulated polar phases such as the dipolar maze and the nano-bubble state have been appraised as essentially distinct. Here we emphasize their topological nature and show that these self-patterned polar states, but also additional mesophases such as the disconnected labyrinthine phase and the mixed bimeron-skyrmion phase, can be fathomed in their plurality through the unifying canvas of phase separation kinetics. Under compressive strain, varying the control parameter, i.e., the external electric field, conditions the nonequilibrium self-assembly of domains, and bridges nucleation and spinodal decomposition via the sequential onset of topological transitions. The evolutive topology of these polar textures is driven by the (re)combination of the elementary topological defects, merons and antimerons, into a plethora of composite topological defects such as the fourfold junctions, the bimeron and the target skyrmion. Moreover, we demonstrate that these manipulable defects are stable at room temperature and feature enhanced functionalities, appealing for devising future topological-based nanoelectronics.

## Abstract (web summary)

Understanding the processes underlying the self-assembly of polar textures is pivotal for the development of future technologies. Here, the authors reveal the dynamics of nonequilibrium phase transitions in low-dimensional ferroelectrics and emphasize their topological nature.

## Introduction

Driven by complex relaxation dynamics, nonequilibrium phenomena such as self-assembly^1^, pattern formation^2^, and phase ordering kinetics^3^ endow phase-separating systems with incomparable richness of domain morphologies. This is particularly relevant in the case of ferroic materials, where complex domain wall arrangements primarily drive emergent phenomena^4^. Understanding the intricate formation processes at play in the formation of modulated phases is thus pivotal for the development of future technologies, e.g., domain wall nanoelectronics^4–20,21,22^, a field of research that has recently seen fiery surge of interest. So far, modulated phases of ferroelectric domains such as the dipolar maze or labyrinthine phase^23^, and the nano-bubble or skyrmionic phase^21,22,24^ have been somewhat regarded as conceptually disparate^24–30^. We here numerically predict and experimentally evidence that, depending on the magnitude of the external field, temperature and the kinetics of the phase separation, topologically non-trivial phases emerge upon sub-critically quenching tetragonal Pb(Zr_*x*_Ti_1 − *x*_)O_3_ through either spinodal decomposition or nucleation processes. The resulting modulated phases are shown to harbor a variety of composite polar topological defects, such as the target skyrmion and the so-called bimeron, that emerge from different combinations of elementary defects. We also show that the self-assembled dipolar patterns, including the yet unreported disconnected labyrinthine and mixed bimerons-skyrmions phases, can be rationalized in their plurality through the unifying canvas of phase separation kinetics. This enables the treatment of chemically homogeneous low-dimensional and elastically constrained ferroelectrics as electrically manipulable phase-separating systems. We also show that the electric field control of skyrmions density elicits hysteretic behavior of conductance, a property that can be harnessed for solid-state neuromorphic computing^31^. These results indicate that the coherent nanoscale intergrowth of topological orders leveraged thus far in diverse materials ranging from metallic alloys^32,33^ to liquid crystals^34^ and polymers^35,36^, can be engineered in ferroic systems as well to enhance their functional topological-based properties.

## Results

We investigate Pb(Zr_0.4_Ti_0.6_)O_3_ (PZT) ultra-thin films through extensive Monte Carlo and molecular dynamics effective Hamiltonian simulations^11,37,38^ (see Supplementary information). The quasi-two-dimensional film is mimicked by an 80 × 80 × 5 supercell (2 nm thickness) subjected to compressive misfit strain of −2.65% and to realistic electric screening at its bottom and top interfaces (screening of 80% of the surface bound charges). Due to the compressive strain that introduces crystalline anisotropy^11,39^, dipoles split into two non-necessarily equal subsets of dipoles pointing normally to the film surface, either upwards (dipole-up) or downwards (dipole-down); the cubic symmetry is reduced to a quasi-${{\mathbb{Z}}}_{2}$, Ising-like symmetry. The concentration of one or the other subset defines the composition of the dipolar binary mixture. This dipolar composition is conditioned by electric boundary conditions, and can be readily tuned through, for example, the application of a homogeneous external electric field perpendicularly to the film plane. Naturally, whenever both dipolar "species” are present, the dipolar composition, the external electric field, as well as the out-of-plane component of polarization can be put in correspondence. This simple observation allows to apprehend the nonequilibrium physics of elastically constrained ultra-thin ferroelectric films by bridging it to chemical phase ordering kinetics of binary mixtures^3,40^.

### Nucleation and spinodal decomposition

**Fig. 1:**
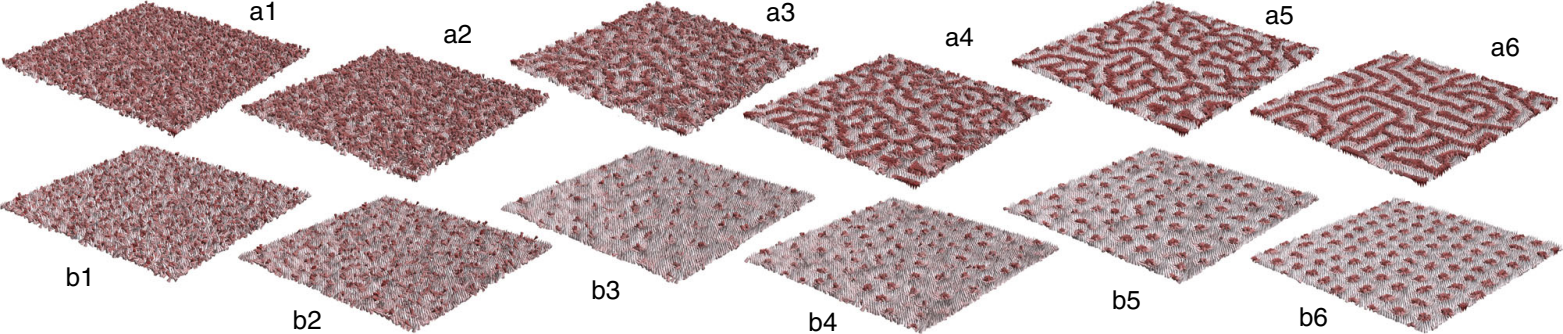
Spinodal decomposition and nucleation processes in PZT thin films. Time evolution of the dipolar pattern within the middle layer of 80 × 80 × 5 film of Pb(Zr_0.4_Ti_0.6_)O_3_ under zero external field (**a**1–**a**6) and under 40 × 10^7^ V/m applied along [001] pseudo-cubic direction (**b**1–**b**6), as obtained from molecular dynamics simulations. In each of the two cases, 1–6 correspond to 150, 250, 350, 450, 750, and 1500 fs after the quench from 650 K to 10 K, respectively. While **a**1–**a**6 indicate the onset of the spinodal instability and development of the labyrinthine pattern, **b**1–**b**6 show the nucleation process leading to the bubble domain pattern. Gray (red) dipoles are oriented along [001] ([00$\bar{1}$]) pseudo-cubic direction.

One route for obtaining a nonequilibrium state is to subject the system to a sudden change in temperature preventing its progressive thermalization, such that its state instantaneously after the change departs from equilibrium under the new conditions^40^. The approach to a new equilibrium state involves collective and complex nonequilibrium processes whereby the single-phase mixture of dipole-up and dipole-down transforms into an inhomogenous and spatially modulated state with coexistence of the two phases separated by domain walls. The emergent self-patterned structures are stabilized by competing long-ranged dipolar interactions and localized ones^39^. In Fig. 1, we show the relaxation with time of two different dipolar configurations: both initial states were thermalized at 650 K, either in the absence of external electric field (with resulting equal dipole-up and dipole-down subsets due to depolarizing field) or under a field value of 40 × 10^7^ V/m (corresponding to a dipole-up subset approximately three times larger than the dipole-down subset), priorly to being sub-critically quenched to 10 K, under zero field and 40 × 10^7^ V/m, respectively. We find that the decay towards equilibrium proceeds along distinctly different pathways depending on the composition of the dipolar binary mixture, that is, on the value of the external field that determines it in average. In the absence of an external field, the homogeneous dipolar binary mixture separates through spinodal decomposition as result of the growth of density fluctuations^40^. As time elapses, concentration gradients further develop and coarsen continuously, resulting in a bicontinuous labyrinthine domain structure consisting of meandering and interconnected domains with sharp interfaces (see Fig. 1a1–a6). Interestingly, the energy of the labyrinth structure is in average only 0.6% higher than that of the parallel stripes domains (which form the ground state of our investigated system), pointing to nearly equally effective energy minimization approach driving the emergence of each of these patterns. The ordering process subtending the results shown in Fig. 1a1–a6 can be substantiated by the analysis of the time dependence of the structure factor after quenching from 650 K to 10 K. We compute the growth rate of fluctuations and, consistently with a common feature of spinodal decomposition^40^, find that all modulations with wavelength *λ*_*c*_ > 1.76 nm (4.42 unit cells) exponentially grow, but that the maximum growth rate is for *λ*_max_ = 3.26 nm (8.16 unit cells), which corresponds to the emergence of a characteristic length in the growth process, the domain width (see Supplementary information). In contrast with such long-wavelength instabilities typical of an initially unstable state, in the presence of an external field, the system evolves through nucleation process^40,41^. The latter is characteristic of the evolution towards equilibrium of an initially metastable state and proceeds via localized composition fluctuations, leading to the formation of bubbles (see Fig. 1b1–b6).

We find that the energy of these spherical droplets has a maximum at a critical radius *R*_*c*_ = 1.4 nm, resulting in the dissolution of droplets with *R* < *R*_*c*_ (for which surface effects are dominant) and the spontaneous growth of those with *R* > *R*_*c*_ (for which volume effects override surface contributions). In addition to this thermal barrier commonly observed in nucleation processes, we find that the depolarizing field induces an absolute minimum, thereby setting an equilibrium radius *R*_eq_ ~1.8 nm for bubbles and hindering their further growth. We note that both *λ*_max_ and *R*_eq_ are reminiscent of the underlying period of the ground-state parallel stripe domain structure (see Supplementary information).

### Sequential topological transitions

**Fig. 2:**
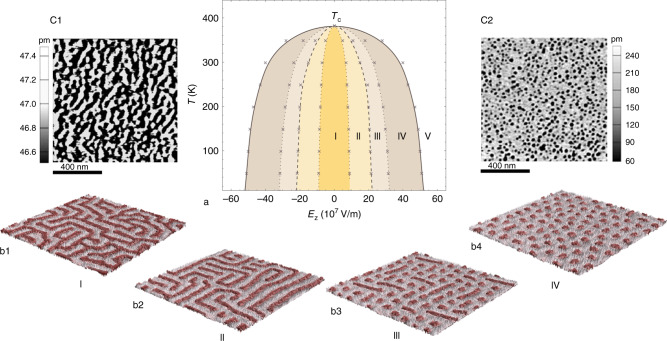
Nonequilibrium phase diagram of compressively strained low-dimensional PZT ferroelectric. **a** Temperature-electric field phase diagram of a Pb(Zr_0.4_Ti_0.6_)O_3_ ultra-thin film mimicked by a 80 × 80 × 5 supercell. Crosses correspond to transition field values calculated through the zeroth Betti number (see Supplementary information) and lines interpolate between the data points obtained for 10 K and from 50 K to 350 K by steps of 50 K. Phases I and II correspond to connected and disconnected labyrinthine patterns, respectively, while phases III and IV denote the mixed bimerons-skyrmion phase and the skyrmions (or bubble) phase, respectively. Phase V corresponds to states where all dipoles are aligned along the applied field. The dashed line separating phases II from III marks the spinodal-like boundary, while the solid line separating phases IV from V marks the binodal-like boundary. **b**1–**b**4 provide dipolar configurations as obtained from simulations within each of phases I–IV in the middle layer of the 80 × 80 × 5 film at 10 K, where gray (red) dipoles are oriented along [001] ([00$\bar{1}$]) pseudo-cubic direction. **c**1, **c**2 show experimental PFM amplitude images of PZT films with 1 unit cells STO spacer and reveal labyrinthine and bubble-skyrmion morphologies, respectively. The labyrinth domain pattern was obtained after annealing the as-grown PZT films at 525 K for 10 min in air, and cooling it down to room temperature at cooling rate of 10–15 K/min. The bubble domains were then created by scanning the labyrinth domains using a SPM probe with an AC amplitude of 500 mV.

Our results further indicate that the transition from the decay of unstable states (via spinodal decomposition) to the decay of metastable states (via nucleation) is gradual and entails the sequential onset of topologically distinct phases with reduced domain connectivity. Notably, identical sequence of domain morphologies can be also observed upon subjecting the labyrinthine pattern to a progressively increasing external field (see Supplementary information). This evolutive topology can be captured by the temperature versus external electric field phase diagram of a PZT ultra-thin film. Different phases are identified through the computation of the zeroth Betti number^42^ for an ensemble of configurations resulting from the field treatment of multiple labyrinthine pattern realizations. This topological invariant, naturally independent of the size, boundary roughness, and shape of the domain wall pattern is associated with the number of connected components, that is the number of heterophase inclusions or domains. The resulting phase diagram is symmetric around the critical point *T*_*c*_ and is composed of nested domes with two curves of special interest. The coexistence curve (solid line in Fig. 2a) corresponds to the outer boundary of the dome structure and delimits the regions where phase separation can occur (phases I–IV) from the homogeneous polar phase (phase V), latter excluding the temperature axis above *T*_*c*_ where the system is paralectric. The spinodal curve (dashed line in Fig. 2a) separates unstable (phases I and II) from metastable (phases III and IV) regions of the phase diagram, where the decay to equilibrium proceeds via spinodal decomposition and nucleation, respectively. The coexistence and spinodal curves meet at *T*_*c*_, temperature characterizing the transition to the parallel stripes ground state as obtained through slowly cooling the system (see Supplementary information). While phase I is associated with the bicontinuous and percolating labyrinthine domain pattern (Fig. 2b1), phase II consists of many meandering and self-avoiding stripes with an overall disconnected labyrinth domain morphology (Fig. 2b2). Within phase III, the domain structure consists of bubbles and straight domains (stripe segments) oriented either along [100] or [010] pseudo-cubic directions (Fig. 2b3). These segments disappear upon reaching phase IV, solely composed of bubbles (Fig. 2b4).

Seeking to ascertain these predictions, we perform piezoresponse force microscopy (PFM) experiments and find striking resemblance between experimental (shown in Fig. 2c1, c2) and numerical data. The experimental results were acquired on an (001)-oriented epitaxial PbZr_0.2_Ti_0.8_O_3_/SrTiO_3_/Pb_0.2_Zr_0.8_TiO_3_ sandwiched films on an La_0.67_Sr_0.33_MnO_3_ buffered SrTiO_3_ (001) substrate. The thicknesses of each PZT layer and SrTiO_3_ (STO) are seven unit cells (u.c.) (~3 nm) and 1 u.c., respectively. The thin film samples were fabricated by pulsed laser deposition, details about the deposition and the as-grown films can be found elsewhere^14^. After having been annealed at 525 K for 10 min in air, the films were cooled down to room temperature at cooling rate of 10–15 K/min, yielding labyrinthine domains (Fig. 2c1). Then, when applying an electric field using the scanning probe microscopy (SPM) under an AC amplitude, the labyrinthine domain pattern starts disappearing (see Supplementary information), ultimately yielding bubble domains at 500 mV (Fig. 2c2).

**Fig. 3:**
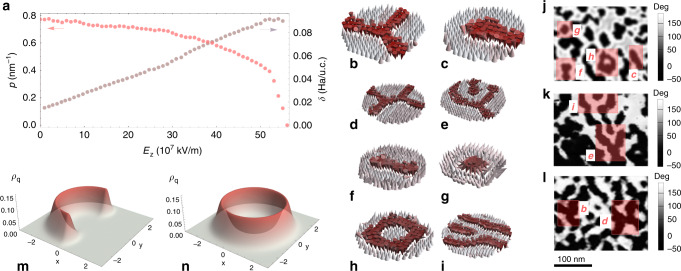
Energetics and topology of domain patterns. **a** Purple curve corresponds to the evolution with increasing field magnitude of the excess interfacial energy at 10 K, *δ*, taken as the difference between average energy of unit cells located at domain walls and unit cells belonging to the minority phase domains. Red curve corresponds to the evolution with increasing field magnitude of the average interfacial length, or perimeter *p*. **b**, **c** show the elementary topological defects within the predicted dipolar modulated phases in the middle layer of Pb(Zr_0.4_Ti_0.6_)O_3_ 80 × 80 × 5 thin film at 10 K, namely, the concave disclination (threefold junction or antimeron) and the convex disclination (meron), respectively. Different combinations of these elementary defects lead to composite defects such as **d** the saddle or fourfold junction (superposition of two concave disclinations), **e** the handle (pair of nearby concave and convex disclinations), **f** the bimeron (elongated skyrmion or superposition of two convex disclinations), **g** the skyrmion, **h** the target skyrmion, and **i** the dislocation. **j**, **k** show experimental PFM phase images of PZT films with 1 unit cells STO spacer. Domain structures were acquired after (**j**) scanning the as-grown area using a SPM probe for 7 times under an external DC bias of +1.8 V and an AC bias of 200 mV, **k** scanning the as-grown area under an external DC bias of +1.5 V once followed by scanning it under an AC bias of 200 mV, **l** scanning the as-grown area using a SPM probe for 8 times under an external DC bias of +1.8 V and an AC bias of 200 mV. Elementary and composite defects are highlighted with red rectangles labeled in correspondence with the numerically predicted defects. **m**, **n** show the Pontryagin density *ρ*_*q*_ as computed from interpolated dipolar vector field for the meron and skyrmion, respectively. The integrated charge yields +1/2 for the meron and +1 for the skyrmion.

Upon traversing the phase diagram horizontally, one can indeed note a gradual loss of connectivity of the domain structure. We find that the mainspring of this gradual crossover from the entirely connected spinodal regime (phase I) to the totally disconnected nucleation regime (phase IV) through transient phases (phases II and III) can be ascribed to the increase in interfacial excess energy of domain walls with increasing field magnitude, as shown in Fig. 3a. This surface tension effect constrains domains towards adopting minimal surface area morphologies, which in turn reflects in the lessening of the interfacial length, or domain wall perimeter, and a gradual disconnection of the domain pattern with increasing field magnitude (also shown in Fig. 3a). We further note that this progressive transition from spinodal decomposition to nucleation contrasts with mean-field expectations of a sharp boundary separating the two^40,41,43^. This ensues from the strong fluctuations to which the ultra-thin ferroelectric film, being a quasi-two dimensional system, is particularly prone to (see Supplementary information). However, these geometrically enhanced fluctuations do not hamper the topological stability of the domain pattern of each of the phases I–IV (as indicated by the constancy of the Betti number within each of these phases, see Supplementary information). Due to the uniformity of topological invariants in between phase boundaries, the field-induced variation of composition within phases is thus achieved by domains adjusting their width without repercussion on their topology. This observation impels us to a topological interpretation^3,39,44^ of the nonequilibrium phase diagram, wherein each phase boundary is also the locus of a topological transition.

### Evolutive topology of elementary and composite defects

**Fig. 4:**
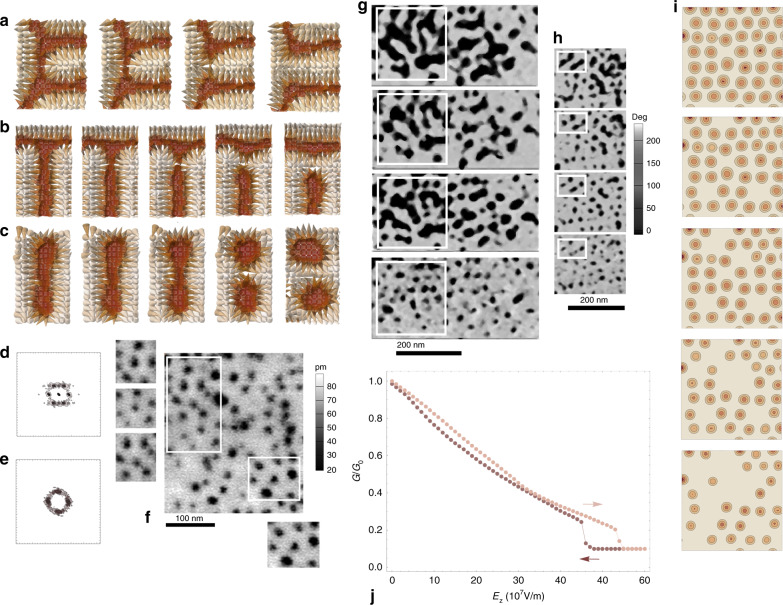
Topological transitional mechanisms and history dependence. Numerical onsets at 10 K of the disconnection processes of domains via the removal of a **a** fourfold junction, **b** threefold junction, and **c** the cleavage of an elongated bimeron into two skyrmions. For each division process, panels correspond to consecutive snapshots of the middle layer of Pb(Zr_0.4_Ti_0.6_)O_3_ 80 × 80 × 5 thin film obtained upon increasing field magnitude at 10 K. **d**, **e** show the calculated structure factors at 10 K of the labyrinthine domain pattern (phase I) and of the skyrmion lattice (phase IV). **f** Experimental evidence at room temperature of the quasi-hexagonal arrangement of polar skyrmions as can be seen in PFM amplitude images of the PZT sandwich thin film. **g**, **h** show experimental realizations of the pattern evolution involving fourfold and threefold, and bimeron, respectively, at room temperature. Figures correspond to PFM phase images where dark to bright contrast indicates a 180° phase difference, and were obtained through successive scans under a DC bias of 1.8 V. **i** Progressive depletion of the skyrmions lattice upon increasing of the field within the range of values triggering the transition from phases IV–V, i.e., the transition from the skyrmions lattice to the monodomain state at 10 K. Bright to dark colors correspond to increasing the out-of-plane component of local dipoles. **j** Electric field hysteresis of normalized tunneling conductance *G*/*G*_0_ at 10 K. The reference tunneling conductance *G*_0_ corresponds to the zero-field labyrinthine state.

Through the distribution of the Pontryagin charge density^45–47^ (the integral of which corresponds to the number of times a unit sphere is wrapped around by the order parameter, i.e., the winding number of polarization), we identify two elementary point topological defects (see Fig. 3b, c), namely the threefold junctions (or concave disclinations of −1/2 charge) and the stripe end-points (or convex disclinations of +1/2 charge, see Fig. 3k) and find that they can differently combine, yielding a plethora of composite defects^48^. We note that the end-points (threefold junctions) can be referred to as convex (concave) disclinations in liquid crystal terminology, or as merons (antimerons) in quantum field theory terms^49^, later borrowed by the magnetic community^50^. The labyrinthine self-patterned domain structure (phase I) harbors many concave and convex disclinations but also, for example, saddle defects (superposition of two concave disclinations), handle defects (pair of nearby concave and convex disclinations or meron-antimeron pair), bimerons, skyrmions, and rare instances of target skyrmions^51^ and dislocations (see Fig. 3b–i for the numerical predictions of defects and Fig. 3j–l for the corresponding experimental realizations). Upon reaching phase II, the pattern loses its junctions (see Fig. 4a, b for fourfold and threefold junctions disconnections, respectively) and as a result, naturally features numerous end-points or merons. Junctions can thus be seen as the weak links of the structure as they are first to disappear. The junction instability roots in a lesser overcompensation of the cost associated with short-range interactions by the gain in electrostatic energy when compared to the energy balance featured by merons. Interestingly, we find that these interactions are sensitive to the local geometry of domains—electrostatic energy is significantly reduced at stripe end-points, where curvature of the surrounding domain wall is extremal. Further, increasing the field results in the nucleation of compact and elongated bimerons (straight stripe segments) within phase III. Both these entities are topologically equivalent to a skyrmion (charge +1, see Fig. 3l). In phase IV, and as a result of a symmetric fission process of elongated bimerons (see Fig. 4c), the pattern is left with only compact bimerons, or skyrmions, in its wake. This last process is akin to what could be seen as a topological mitotic division, with the appearance of a cleavage furrow whose development entails greater concavity, and through which the topological inheritance is transmitted (an elongated bimeron is identically charged to the two newly created skyrmions). Phase IV can thus be deemed as being a polar skyrmionic phase, endowed with a quasi-crystalline weak hexagonal symmetry (see Fig. 4d), in contrast with the nearly square symmetry (structure factor giving the appearance of a four-peaked crown^39^, see Fig. 4e) of the labyrinth phase (or phase I), latter being reminiscent of the lattice geometry. Consistently with these topological insights, we experimentally witness occurrences of hexagonal arrangements of bubble-skyrmion domains (see Fig. 4f) and also find the imprint of transitional domain topologies (Fig. 4g, h). The corresponding PFM images were obtained through successive scans under an external electric field of 25 × 10^7^ V/m (which corresponds to a DC voltage of 1.8 V) applied via the conductive probe-tip at room temperature. Note that numerically, these transitions occur for field values ranging from  ~10 × 10^7^ V/m to 30 × 10^7^ V/m. Interestingly, for field values approaching the transition line between phases IV and V, the skyrmion lattice starts depleting in a discrete manner, as if it was undergoing a sublimation process where skyrmions detach from the solid phase to form a gas in the empty space (see Fig. 4i). This particle-like behavior can be ascribed to the topological protection of skyrmions, which is lifted whenever the energy injected by the external field surmounts the topological energy barrier.

### Hysteretic behavior of tunneling conductance

Additionally, this transition to the monodomain state exhibits hysteretic behavior associated with the dependence of the z-component of polarization on the external electric field. Such history dependent behavior can be leveraged to devise memristor-based solid-state synapses for unsupervised machine learning circuits^31^. Further confirmation of the memristor behavior is provided by an estimate of the tunneling conductance dependence on the external field magnitude (see Fig. 4j). It can be seen that the upper branch of this dependence corresponding to switching towards the monodomain state upon increasing field magnitude exhibits up to  ~250% higher conductance than that of the inverse-path switching branch. Furthermore, one can note that the mild switching slopes, unusual in such ferroelectric systems, can allow for fine conductance tuning. Interestingly, weaker hysteretic behavior is seen for all lower field values, which suggests a complex hierarchical structure of the free-energy landscape of the system (see Supplementary information).

In summary, we report on the dynamics of nonequilibrium phase transitions in low-dimensional ferroelectrics and emphasize their topological nature. We analyze the self-patterning of domains within the unifying picture of phase separation kinetics for a wide range of external electric field magnitudes and temperatures, and demonstrate that the presence of an unstable mode entails the onset of a labyrinthine phase through spinodal decomposition, while that of a metastable mode reflects in the nucleation of a quasi-hexagonal skyrmion lattice phase. Furthermore, a departure from mean-field expectations reveals additional polar mesophases and highlights the various topological instabilities domains are prone to at transitions. The self-assembly of domains is shown to yield composite polar topological defects, such as the target skyrmion and bimeron, that were thus far eluded in low-dimensional ferroelectrics. Our experimental data supports the view that the field-induced transitions are predicated on the prominent role of topological defects, and further demonstrates that their reversible manipulation at room temperature and on a nanometric scale is readily achievable. These results, including the hysteresis of conductance associated with the creation and annihilation of skyrmions, render the behavior of these patterns appealing for devising future nanoelectronics.

## Methods

### Computational details

We mimic Pb(Zr_0.4_Ti_0.6_)O_3_ (PZT) ferroelectric ultra-thin films that are grown along the [001] direction (which is chosen to be the *z*-axis) and are Pb-O terminated at all surfaces/interfaces. Note that the local modes are centered on the B-sites and the film terminates by an A-site Pb-O layer. The studied films typically have a thickness of 2.0 nm (i.e., of 5 unit cells), and are subjected to a compressive strain of −2.65% in order to ensure that dipoles have a preferential direction along the out-of-plane direction. Such value would approximately account for the mismatch of lattice constants of the cubic phases of strontium titanate (STO) and PZT. They are interposed between (realistic) electrodes that can only screen 80% of the polarization-induced surface charges, and modeled by various *L* × *L* × 5 supercells that are all periodic along the [100] and [010] directions while finite along the *z*-axis. Technically, a first-principles-based effective Hamiltonian^21^ is used within Monte Carlo simulations to determine the energetics and local electric dipoles in each perovskite five-atom cell of these supercells. Models parameters are given in ref. ^9^ The validity of this approach was demonstrated by previous theoretical studies of ultra-thin PZT films under compressive strains that (1) yield 180 degrees “up” and “down” stripe domains that periodically alternate along [100] (or along [010]) for their ground state^11,21^, in agreement with experimental observation^12^ (note that “up” (respectively, “down”) domains refer to domains in which the z-component of the dipole is parallel (respectively, antiparallel) to the *z*-axis, respectively); (2) predict a linear dependency between the width of these periodic stripes and the square root of the film’s thickness^52^, as consistent with measurements^53^; and (3) have also led to the prediction of various topological defects such as vortices^8^, dipolar waves^54^, bubbles^21^, and merons (or convex disclinations)^13^ in ferroelectrics, that have been experimentally confirmed^13,14,55^. Note that the predicted temperature has to be rescaled by a factor of  ~1.6 with respect to measurements^56^.

The phase diagram presented in Fig. 2 of the manuscript is obtained in the following manner: for each of the considered temperatures, we perform 200 independent zero-field thermal quenches and then subject the obtained configurations to an electric field rising up to 60 × 10^7^ V/m with steps of 1 × 10^7^ V/m. This allows to obtain the field-induced evolution of the average zeroth Betti number, whose logarithmic derivative exhibits local extrema at transitions between distinct domain topologies.

### Experimental details

Experimental results were acquired on a (001)-oriented epitaxial PbZr_0.2_Ti_0.8_O_3_/SrTiO_3_/PbZr_0.2_Ti_0.8_O_3_ sandwich films (PZT/STO/PZT) on a 15 nm thick La_0.67_Sr_0.33_MnO_3_(LSMO) buffered STO (001)substrate. The thickness of each PZT layer and STO spacer layer are 7 unit cells (u.c.) (~3 nm) and 1 u.c., respectively. The thin film samples were fabricated by pulsed laser deposition, the deposition details can be found elsewhere^14^. Nanoscale bubble domains with lateral domain size of <10 nm are observed homogenously distributed in the as-grown PZT sandwich films at room temperature. The experimental evidence of domain transition was visualized by PFM images, which were acquired by using a commercial SPM (Cypher S, Asylum Research, USA). Conductive Cr/Pt-coated silicon cantilevers (BudgetSensors ElectricMulti75-G, Bulgaria) were used for experimental domain imaging under an AC amplitude ranging from 200 to 500 mV. The labyrinth domains occurred after annealing the films on a hot plate at 525 K for 10 min in air followed by cooling to room temperature at cooling rate of 10–15 K. Note that, previously, we had experimentally demonstrated the transition from an as-grown bubble state to labyrinth state upon the application of an electric field^14^, while in this study, we show that the labyrinth domain pattern can be further obtained through the temperature treatment described above. The transition between the heat treatment-derived labyrinth domains to bubble domain states was realized by applying an AC bias (500 mV) via SPM probe during scanning. The evidence for the topological evolution between labyrinth and bubble domains was acquired via SPM by scanning the films under a DC bias (voltages were applied via film bottom electrode towards the scanning probe) of +1.8 V with a reading AC bias of 200–300 mV. All SPM experiment were conducted at room temperature.

## Supplementary information

Supplementary Information

## Data Availability

The relevant data sets generated during and/or analyzed during the current study are available from the corresponding authors on reasonable request.

## References

[1] Desai RC, Kapral R (2009). Dynamics of Self-organized and Self-assembled Structures.

[2] Cross MC, Hohenberg PC (1993). Pattern formation outside of equilibrium. Rev. Mod. Phys..

[3] Bray AJ (1994). Theory of phase ordering kinetics. Adv. Phys..

[4] Catalan G, Seidel J, Ramesh R, Scott JF (2012). Domain wall nanoelectronics. Rev. Mod. Phys..

[5] Salje EKH (2010). Multiferroic domain boundaries as active memory devices: trajectories towards domain boundary engineering. Chem. Phys. Chem..

[6] Scott JF, Paz de Araujo CA (1989). Ferroelectric memories. Science.

[7] QuanJiang A (2015). Giant dielectric permittivity in ferroelectric thin films: domain wall ping pong. Sci. Rep..

[8] Naumov II, Bellaiche L, Fu H (2004). Unusual phase transitions in ferroelectric nanodisks and nanorods. Nature.

[9] Garcia V (2009). Giant tunnel electroresistance for non-destructive readout of ferroelectric states. Nature.

[10] Kim DJ (2012). Ferroelectric tunnel memristor. Nano Lett..

[11] Kornev IA, Fu H, Bellaiche L (2004). Ultrathin films of ferroelectric solid solutions under a residual depolarizing field. Phys. Rev. Lett..

[12] Streiffer SK (2002). Observation of nanoscale 180 degrees stripe domains in ferroelectric PbTiO_3_ thin films. Phys. Rev. Lett..

[13] Lu L (2018). Topological defects with distinct dipole configurations in PbTiO_3_-SrTiO_3_ multilayer films. Phys. Rev. Lett..

[14] Zhang Q (2017). Nanoscale bubble domains and topological transitions in ultrathin ferroelectric films. Adv. Mater..

[15] Gu Z (2018). Resonant domain-wall-enhanced tunable microwave ferroelectrics. Nature.

[16] Hlinka J, Paściak M, Körbel S, Marton P (2017). Terahertz-range polar modes in domain-engineered BiFeO_3_. Phys. Rev. Lett..

[17] Xue F, Ji YZ, Chen LQ (2017). Theory of strain phase separation and strain spinodal: applications to ferroelastic and ferroelectric systems. Acta Mater..

[18] Balke N (2012). Enhanced electric conductivity at ferroelectric vortex cores in BiFeO_3_. Nat. Phys..

[19] Sharma P (2019). Conformational domain wall switch. Adv. Funct. Mater..

[20] Zhang Q (2019). Deterministic switching of ferroelectric bubble nanodomains. Adv. Func. Mater..

[21] Lai B-K (2006). Electric-field-induced domain evolution in ferroelectric ultrathin films. Phys. Rev. Lett..

[22] Das. S (2019). Observation of room-temperature polar skyrmions. Nature.

[23] Nahas Y (2020). Inverse transition of labyrinthine domain patterns in ferroelectric thin films. Nature.

[24] Hong Z, Chen LQ (2018). Blowing polar skyrmion bubbles in oxide superlattices. Acta Mater..

[25] Eliseev EA (2018). Labyrinthine domains in ferroelectric nanoparticles: manifestation of a gradient-induced morphological transition. Phys. Rev. B.

[26] Ahluwalia R, Cao W (2000). Influence of dipolar defects on switching behavior in ferroelectrics. Phys. Rev. B.

[27] Ricinschi D, Okuyama M (2007). Field-dependent switching kinetics and ferroelectric hysteresis loops analyzed with a phenomenological model in relation to typical experiments. Ferroelectrics.

[28] Artemev A, Roytburd A (2010). Spinodal single to polydomain transition and P-E hysteresis in thin ferroelectric films. Acta Materi..

[29] Gerra G, Tagantsev AK, Setter N (2005). Surface-stimulated nucleation of reverse domains in ferroelectrics. Phys. Rev. Lett..

[30] Burton BP, Nishimatsu T (2007). First principles phase diagram calculations for the system NaNbO_3_-KNbO_3_: can spinodal decomposition generate relaxor ferroelectricity?. Appl. Phys. Lett..

[31] Boyn S (2017). Learning through ferroelectric domain dynamics in solid-state synapses. Nat. Commun..

[32] Kato M, Mori T, Schwartz LH (1980). Hardening by spinodal modulated structure. Acta Met..

[33] Kim MU (2009). Applications of spinodal decomposition to produce metallic glass matrix composite with simultaneous improvement of strength and plasticity. Met. Mater. Int..

[34] Nagaya T, Gilli JM (2002). Experimental study of coarsening dynamics of the zigzag wall in a nematic liquid crystal with negative dielectric anisotropy. Phys. Rev. E.

[35] Nisato G, Ermi BD, Douglas JF, Kari A (1999). Excitation of surface deformation modes of a phase-separating polymer blend on a patterned substrate. Macromolecules.

[36] Sehgal A, Ferreiro V, Douglas JF, Amis EJ, Karim A (2002). Pattern-directed dewetting of ultrathin polymer films. Langmuir.

[37] Ponomareva I, Naumov II, Bellaiche L (2005). Low-dimensional ferroelectrics under different electrical and mechanical boundary conditions. Phys. Rev. B.

[38] Ponomareva I, Naumov II, Kornev I, Fu H, Bellaiche L (2005). Atomistic treatment of depolarizing energy and field in ferroelectric nanostructures. Phys. Rev. B.

[39] De’bell K, MacIsaac AB, Whitehead JP (2000). Dipolar effects in magnetic thin films and quasi-two-dimensional systems. Rev. Mod. Phys..

[40] Chaikin, P.M. & Lubensky, T.C. *Principles of Condensed Matter Physics* (Cambridge University Press, Cambridge, UK, 1995).

[41] Binder K (1987). Theory of first-order phase transitions. Rep. Prog. Phys..

[42] Sofonea V, Mecke KR (1999). Morphological characterization of spinodal decomposition kinetics. Eur. Phys. J. B.

[43] Schmelzer JWP, Abyzov AS, Möller J (2004). Nucleation versus spinodal decomposition in phase formation processes in multicomponent solutions. J. Chem. Phys..

[44] Seul M, Andelman D (1995). Domain shapes and patterns: the phenomenology of modulated phases. Science.

[45] Nahas Y (2015). Discovery of stable skyrmionic state in ferroelectric nanocomposites. Nat. Commun..

[46] Prokhorenko S, Nahas Y, Bellaiche L (2017). Fluctuations and topological defects in proper ferroelectric crystals. Phys. Rev. Lett..

[47] Nagaosa N, Tokura Y (2013). Topological properties and dynamics of magnetic skyrmions. Nat. Nanotechnol..

[48] Passot T, Newell AC (1994). Towards a universal theory for natural patterns. Phys. D..

[49] Callan CG, Dashen R, Gross DJ (1978). Toward a theory of the strong interactions. Phys. Rev. D..

[50] Yu XZ (2018). Transformation between meron and skyrmion topological spin textures in a chiral magnet. Nature.

[51] Leonov AO, Rößler UK, Mostovoy M (2014). Target-skyrmions and skyrmion clusters in nanowires of chiral magnets. EPJ Web Conf..

[52] Lai B-K, Ponomareva I, Kornev IA, Bellaiche L, Salamo GJ (2007). Thickness dependency of 180 degree stripe domains in ferroelectric ultrathin films: a first-principles study. Appl. Phys. Lett..

[53] Schilling A (2006). Scaling of domain periodicity with thickness measured in BaTiO_3_ single crystal lamellae and comparison with other ferroics. Phys. Rev. B.

[54] Sichuga D, Bellaiche L (2011). Epitaxial Pb(Zr,Ti)O_3_ ultrathin films under open-circuit electrical boundary conditions. Phys. Rev. Lett..

[55] Yadav AK (2016). Observation of polar vortices in oxide superlattices. Nature.

[56] Bellaiche L, Garcia A2, Vanderbilt D (2000). Finite-temperature properties of Pb(Zr_1−*x*_ Ti_*x*_)O_3_ alloys from first principles. Phys. Rev. Lett..

