# Supplementary material for: Topology and control of self-assembled domain patterns in low-dimensional ferroelectrics
Source: Nat Commun. 2020 Nov 13;11:5779. doi: 10.1038/s41467-020-19519-w (PMC7666159; doi:10.1038/s41467-020-19519-w)
Supplement: Supplementary file 1 — Supplementary Information [file 41467_2020_19519_MOESM1_ESM.pdf]

Supplementary Information: Topology and control of self-assembled domain patterns in low-dimensional ferroelectrics

Y. Nahas[1], S. Prokhorenko[1], Q. Zhang[2], V. Govinden[2], N. Valanoor[2], and L. Bellaiche[1]
[1] *Physics Department and Institute for Nanoscience and Engineering, University of Arkansas, Fayetteville, AR 72701, USA*
[2] *School of Materials Science and Engineering, University of New South Wales, Sydney, New South Wales 2052, Australia*

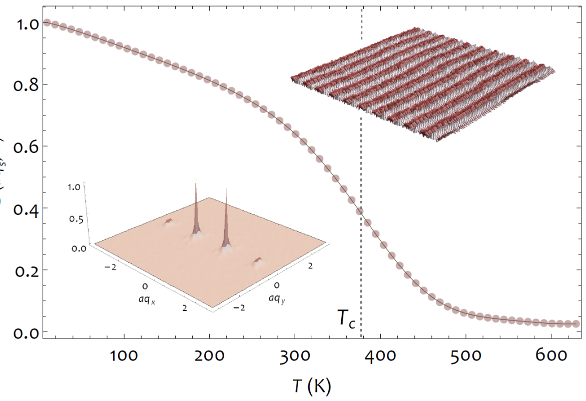

FIG. S1: Temperature variation of the scaled structure factor $\tilde{S}(a\vec{q_s}, T)$ through the slow cooling of a 80×80×5 film of $Pb(Zr_{0.4}Ti_{0.6})O_3$. $\tilde{S}(aq_s, T)$ is taken as the ratio of $S(a\vec{q_s}, T)$ to $S(a\vec{q_s}, 10\text{ K})$, where a is the lattice parameter and $\vec{q_s}$ the $\vec{q}$ point corresponding to the wave length of the striped phase modulation (see right inset showing the dipolar configuration in the middle layer of the film at 10 K where gray (red) dipoles are oriented along [001] ([00$\bar{1}$]) pseudo-cubic direction). $S(a\vec{q}, T)$ is computed as the thermodynamic average of the squared norm of the three-dimensional discrete Fourier transform of the z component of local modes u_z. Left inset shows the structure factor at 10 K. It exhibits two primary peaks along one direction of the principal axes of the Brillouin zone, reflecting the twofold symmetry of the striped phase and its orientational order. Subsidiary peaks are due to finite size effects. Vertical dashed line indicates the inflection point of $\tilde{S}(a\vec{q_s}, T)$ and is taken as the locus of T_c.

In figure S1, we provide the temperature variation of the scaled structure factor $\tilde{S}(aq_s, T)$ upon slowly cooling the system. $\tilde{S}(aq_s, T)$ is taken as the ratio of $S(aq_s, T)$ to $S(aq_s, 10\text{ K})$, a is the lattice parameter and q_s is the q point corresponding to the wave length of the striped phase modulation. $S(aq, T)$ is computed as the thermodynamic average of the squared norm of the three-dimensional discrete Fourier transform of the z component of local dipoles u_z. Looking into the behavior of $\tilde{S}(aq_s, T)$, it is readily seen that its rise below $T_c \sim 380$ K signals the emergence of the parallel stripes modulation. Right and left panels show the obtained ground state configuration and its corresponding structure factor at 10 K.

In panel (a) of figure S2, we provide the wave vector dependence of the circularly averaged structure factor for different times following the quench to 10 K under zero external field, as obtained from Molecular Dynamics (MD) simulations of an 80×80×5 film of $Pb(Zr_{0.4}Ti_{0.6})O_3$. $S(a|q|, t)$ corresponds to the two-dimensional Fourier transform of the z-component of the dipolar field at time t and can be seen as the dynamical correlation function of the concentrational fluctuations of up and down "species" of the dipolar mixture, thus capturing the evolution of phase separation as the system is quenched away from equilibrium. As time elapses, the emergence of order is buttressed by the development of a peak which grows in intensity and moves to lower $a|q|$, reflecting the growth of domains with time. Ultimately, there are no further changes in $S(a|q|, t)$, a freezing behavior that can be ascribed to the electric boundary conditions and film thickness, the two factors that condition the periodicity of the alternating domain pattern. For early times, we observe that $S(a|q|, t)$ behaves as $e^{\alpha(a|q|)t}$. The dependence of this exponential growth rate α on the wave-vector $a|q|$ is shown in panel (b) of figure S2. All modulations with wavelength $\lambda_c > 1.76$ nm (4.42 unit cells) exponentially grow, while those with wavelength inferior to λ_c are damped. The maximum growth rate is for $\lambda_{max} = 3.26$ nm (8.16 unit cells). It is this most rapidly growing mode with maximal amplification rate of decomposition that will dominate and define the characteristic length-scale of the emergent pattern. In the nucleation regime, the thermodynamics of the phase separation process is reflected in the free-energy-like potential $\Delta\tilde{F}/k_BT$ as

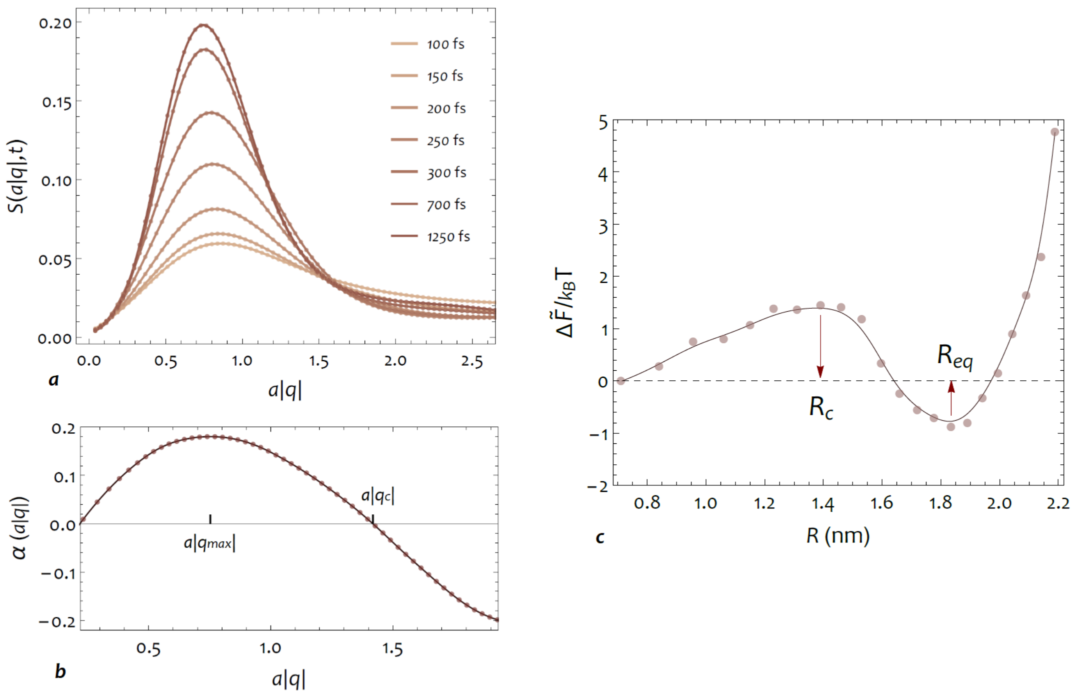

FIG. S2: Panel (a) provides the wave vector dependence of the circularly averaged structure factor for different times following the quench to 10 K under zero external field, as obtained from MD simulations of an 80×80×5 film of $Pb(Zr_{0.4}Ti_{0.6})O_3$. Specifically, $S(a|q|,t) = (1/2\pi)\int_0^{2\pi} d\phi\, \langle S(a|q|\cos\phi, a|q|\sin\phi, t)\rangle$, where $\langle\cdot\rangle$ denotes the thermodynamic average over 100 quenches, and $S(a|q|\cos\phi, a|q|\sin\phi, t)$ corresponds to the two-dimensional Fourier transform of the z-component of the dipolar field at time t. $|q|$ and ϕ are the reciprocal space polar coordinates. It is seen that as time elapses, the peaks sharpens and shifts to lower wave vector values. Panel (b) shows the dependence of α, the growth rate of the spinodal regime, on the wave vector magnitude as extracted from data shown in panel (a). Two special $a|q|$ points, $a|q_{max}|$ and $a|q_c|$ can be seen, and indicate the wave vector magnitudes at which the growth rate of modulations is maximum and zero, respectively. Panel (c) shows the free-energy-like potential $\Delta\tilde{F}/k_BT$ versus the bubble skyrmion radius. $\Delta\tilde{F}$ is obtained from sampling the distribution of skyrmion radii during the nucleation process. Note that $\Delta\tilde{F}$ differs from the accurate out-of-equilibrium free-energy F by an additive constant that depends only on external parameters (e.g. temperature, external electric field). This arbitrariness is however not essential for our purposes. The zero reference value of $\Delta\tilde{F}$ is chosen to correspond to the minimum possible bubble skyrmion domain radius. Solid line is a 10th degree B-spline interpolation of obtained data points. Arrows indicate the critical (R_c) and equilibrium (R_{eq}) bubble skyrmion radii.

a function of the bubble skyrmion radius (panel (c) of figure S2). This function is practically obtained by sampling the skyrmion radii distribution from an ensemble of 50 distinct molecular dynamics trajectories, all obtained upon quenching the system from 650 K down to 10 K under an external field of 40 × 10^7 V/m. For each trajectory, the dipolar structures were collected each 100 fs, overall yielding a sampling set of 100 configurations per trajectory. Each configuration was analyzed through the Hoshen-Kopelman clustering algorithm[1] which allowed to directly identify the volume of each bubble domain and consequently the distribution $\rho(R)$ of bubble radii R. The free-energy like function $\Delta\tilde{F}/k_BT$ is then obtained by taking $-\ln\rho(R)$. Note that the discreteness of the lattice and the employed algorithm impose a minimal value of approximately $R = 0.56$ for the bubble skyrmion radius. We find that a maximum of the free-energy-like potential at a critical radius $R_c = 1.4$ nm, which indicates the dissolution of droplets with $R < R_c$, while for $R > R_c$ the localized heterophase fluctuations spontaneously grow. The growth is however bound by the depolarizing field that imposes an absolute minimum associated with an equilibrium radius $R_{eq} \sim 1.8$ nm for skyrmion bubbles. As in the case of the spinodal decomposition process, we find that the characteristic length scale of patterns is conditioned by the geometry of the film and the electric boundary conditions at play.

In panel (a) of figure S3, we show the logarithm of the probability distribution function ρ of the z-component of the local modes[2] (which are directly proportional to the local electric dipoles) within the 80×80×5 $Pb(Zr_{0.4}Ti_{0.6})O_3$ ultra-thin film, estimated through the slow cooling of the system under zero external field. The curves correspond to the 6th-order polynomial fits (with standard error on parameters below 4%) of ρ distributions that were numerically obtained from 100 statistically independent configurations at each temperature. For each temperature, the statistical sample amounted to 3.2 million u_z values, and the bin width is taken to be of 0.001. Although ρ is computed using

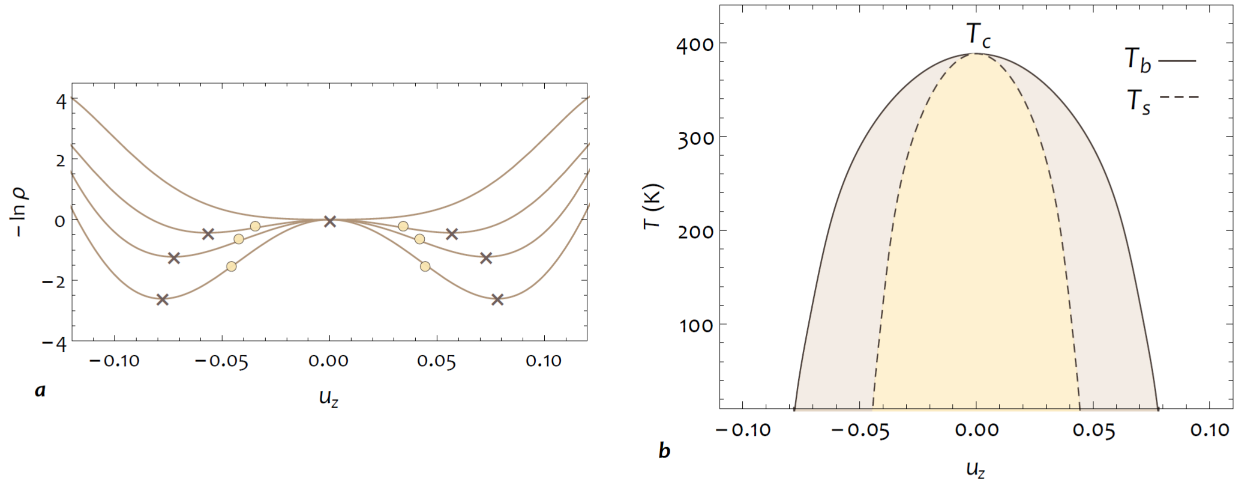

FIG. S3: (a) Logarithm of the probability distribution function ρ of the z-component of the local modes within the 80×80×5 supercell at 10 K, 110 K, 285 K and 385 K. Solid lines are 6th-order polynomial fits of the obtained data points. Crosses indicate the position of the minima and circles denote the inflection points. (b) Mean-field temperature-polarization phase diagram of $Pb(Zr_{0.4}Ti_{0.6})O_3$ ultra-thin film. The phase diagram is composed of two bell-shaped lines, the outer one corresponding to a binodal-like boundary T_b (obtained from the position of the minima of $-\ln\rho$ at each temperature) and the inner one to the spinodal-like boundary T_s (obtained from the position of the inflection points of $-\ln\rho$ at each temperature). The spinodal and binodal curves meet at T_c.

equilibrium configurations obtained from MC simulations thus capturing many-body effects, being a single-variable distribution, it does not describe spatial correlations among dipoles and hence $-\ln\rho$ can be regarded as a mean-field potential. As expected, it is seen that upon increasing temperature, the double well structure gradually flattens, with wells becoming shallower while the distance between equivalent minima decreases, ultimately resulting in a single minimun potential at u_z=0 for $T \geq T_c$. In panel (b) of figure S3, we provide the mean-field-like phase diagram constructed from such mean-field potentials at different temperatures. The outer and inner bell-shaped lines of this phase diagram correspond to a binodal-like boundary T_b (obtained from the position of the minima of $-\ln\rho$ at each temperature) and the spinodal-like boundary T_s (obtained from the position of the inflection points of $-\ln\rho$ at each temperature). For quench temperatures and u_z values falling in-between the binodal and spinodal curves, the system is in metastable equilibrium, and the decay towards the equilibrium state occurs initially via droplet (or bubble skyrmion) nucleation, a hallmark process of first-order phase transitions. Below the spinodal curve, the system is unstable and evolves continuously through spinodal decomposition towards labyrinthine morphologies. For $u_z = 0$, the spinodal and binodal curves meet at T_c, temperature characterizing the equilibrium and continuous transition to parallel stripes obtained through slowly cooling the system.

The universal mapping of the employed effective Hamiltonian model to the general theory of phase separation kinetics can be achieved by expressing the free-energy density of the compressively strained film $\tilde{f}$ with quasi-$\mathbb{Z}_2$ local symmetry in the following form:

$$\tilde{f}(\phi_z) = f(\phi_z) - E_z\phi_z + (1-\beta)\frac{\delta_\infty}{2}P_z\phi_z, \tag{1}$$

where ϕ_z denotes the local z-component of polarization varying with the spatial coordinates x and y within the film plane, $\delta_\infty \approx 4\pi$ is the average depolarizing factor for homogeneously polarized film, while E_z and P_z correspond to the external electric field applied perpendicular to the film surface and the average z-component of the film polarization ($P_z = 1/S\int dxdy\,\phi_z(x,y)$, with S corresponding to the surface area of the film), respectively. The last term in Eq. (1) describes the free-energy contribution stemming from the homogeneous depolarizing field generated by the zero wave-vector ($q = 0$) polarization mode under residual depolarizing field. The physical meaning of the first term in Eq. (1) can be then described in the following way: for $q = 0$ the $f(\phi_z)$ term corresponds to the free-energy of the ferroelectric material under ideal short-circuit boundary conditions, while, for $q \neq 0$, $f(\phi_z)$ differs from its short-circuit counterpart by the inhomogeneous contribution of the depolarizing field energy[3].

Explicitly separating the external electric field and $q = 0$ depolarizing field energy contributions allows to notice that the last two terms in Eq. (1) formally correspond to the constraint $\Lambda\phi_z$ with Lagrange multiplier Λ parametrized as $\Lambda = (1-\beta)\delta_\infty/2P_z - E_z$. Therefore, $\tilde{f}(\phi_z)$ can be seen as the free-energy of the film under the condition of

conserved order parameter $\int dxdy\,(\phi_z(x,y) - P_z) = 0$, where P_z is defined by

$$\left.\frac{\delta f}{\delta \phi_z}\right|_{\phi_z = P_z} = \Lambda. \tag{2}$$

In turn, this observation allows to universally identify Eqs. (1-2) with the general theory of phase separation kinetics[4]. Furthermore, we would like to note that, within the mean-field approximation, one can establish a monotonic mapping of the constrained P_z value to the value of the control parameter E_z, thereby qualitatively explaining the classical, "bell"-like shape[4] of the phase regions in the phase diagram obtained from simulations (see Fig. 2 of the manuscript and Fig. S3). Indeed, taking into account the condition of the stripe-domain ground state granted by the compressive epitaxial strain and partial screening of the bound charges we can write the Landau free-energy $\tilde{f}_{\mathrm{MF}}$ expansion in the following general form:

$$\tilde{f}_{\mathrm{MF}} = \kappa_0 P_z^2 - E_z P_z + \kappa_{\vec{q}_s} Q^2 + \gamma' Q^4 + 2\gamma' Q^2 P_z^2, \tag{3}$$

where Q denotes the magnitude of the polarization mode corresponding to the ground-state domain pattern ($Q = \sqrt{S(a\vec{q}_s)}$). The suppression of $q = 0$ instability by the depolarizing field means that κ_0 is positive for all temperatures, while $\kappa_{\vec{q}_s} \sim (T - T_c)$ (see Fig. S1). Both the quartic Q^4 term as well as the bi-quadratic coupling $Q^2 P_z^2$ stem from the anharmonic on-site energy contributions of the effective Hamiltonian. This can be shown by re-writing the corresponding sum over the lattice dipoles $\sum_i u_i^4$ in reciprocal space. Performing the Fourier transformation and summation of the resulting expression over the lattice sites results in the non-local quartic interaction between the modes with vanishing overall momentum[5,6], i.e.

$$\sum_i u_i^4 \rightarrow \sum_{\vec{q}_1 \vec{q}_2 \vec{q}_3 \vec{q}_4} \delta_{\vec{q}_1 + \vec{q}_2 + \vec{q}_3 + \vec{q}_4 = \vec{0}}\, u_{\vec{q}_1} u_{\vec{q}_2} u_{\vec{q}_3} u_{\vec{q}_4}. \tag{4}$$

Considering only the $\vec{q} = \vec{0}$ and $\vec{q} = \pm\vec{q}_s$ modes in Eq. (4) (e.g. $\vec{q}_1 = \vec{q}_2 = 0$; $\vec{q}_3 = \vec{q}_s$; $\vec{q}_4 = -\vec{q}_s$ or $\vec{q}_1 = \vec{q}_2 = \vec{q}_s$; $\vec{q}_3 = \vec{q}_4 = -\vec{q}_s$ etc.) we can now confirm the origin of fourth-order terms in Eq.(3). Moreover, by counting the number of possible permutations of wavevector values (six terms for $\vec{q}_{1-4} = \pm q_s$ *versus* twelve terms coupling $\vec{q} = \vec{0}$ and $\vec{q} = \pm\vec{q}_s$ modes) one can also see that the bi-quadratic coupling strength between P_z and Q should be two times larger than the quartic coefficient of the Q^4 term. More generally, one can show that, at low temperatures and for the considered elastic boundary conditions, γ' is proportional to a linear combination $c_1\alpha^{\|} + c_2\alpha^{\perp} + c_3 * \gamma^{\|} + c_4\gamma^{\perp}$, where c_{1-4} are positive constants and

$$\begin{aligned}
\alpha^{\|} &= \alpha - B_{1yy}^2/(2b_{11}) \\
\alpha^{\perp} &= \alpha - B_{1xx}^2/(2b_{11}) \\
\gamma^{\|} &= \gamma + 2\alpha - B_{1yy}^2/b_{11} \\
\gamma^{\perp} &= \gamma + 2\alpha - B_{1xx}B_{1yy}/b_{11} - B_{4yz}^2/(2b_{44})
\end{aligned}$$

In the above expressions α and γ denote the on-site anharmonic coefficients of the effective Hamiltonian model while b and B constants describe elastic and electrostrictive properties of the ferroelectric material, respectively. We note that the κ_0 and $\kappa_{\vec{q}_s}$ coefficients in Eq.(3) can be also generally linked to the microscopic model parameters. However, in contrast to 4-th order expansion coefficients, one can expect a significant renormalization of these terms by thermal fluctuations.

Minimizing $\tilde{f}_{\mathrm{MF}}$ with respect to P_z and Q results in

$$P_z = \frac{E_z}{2(\kappa_0 + 2\gamma' Q^2)} \tag{5}$$

and shows that Q monotonically decreases upon increasing electric field magnitude. This result establishes a monotonic correspondence between P_z and E_z. From Eqs. (5,3) one can also see that the electric susceptibility at zero-field is equal to $\chi|_{E_z=0} = 1/2(\kappa_0 - \kappa_{\vec{q}_s})$. Building on this observation, one can estimate the values of κ_0 and $\kappa_{\vec{q}_s}$ from the temperature dependencies of Q and the zero-field susceptibility.

Interestingly, Eq.(3) also allows us to estimate the field magnitude at which the system transits to the monodomain state. Expanding the free-energy difference between the $Q \neq 0$ and $Q = 0$ states in powers of Q in the vicinity of this transition line, where $Q\sqrt{-2\gamma'/\kappa_{\vec{q}_s}} \ll 1$, and requiring the leading order expansion coefficient to be equal to zero yield the critical field value of

$$E_z^{\text{IV-V}} = \kappa_0 \sqrt{-2\kappa_{\vec{q}_s}/\gamma'}. \tag{6}$$

Notably this result links to the general theory of phase separation kinetics. Indeed, as it can be seen from Eq.(5), the value of polarization at $E_z^{\text{IV-V}}$ is equal to $E_z^{\text{IV-V}}/2\kappa_0$ or, equivalently

$$P_z^{\text{IV-V}} = \sqrt{-\kappa_{\vec{q_s}}/2\gamma'}. \tag{7}$$

This expression can be readily recognized as the equilibrium value of the order parameter Q at zero external field, which, by definition of Q, corresponds to the position of the minimum of the $f(\phi_z)$ (see also the binodal line in Fig. S3).

The position of the spinodal line separating phases II and III corresponds to the inflection point of the $f(\phi_z)$ potential. From the discussion presented above it follows that an estimate of the corresponding transition field value can be obtained by identifying the position of the inflection point of the Landau free-energy of homogeneously polarized film under ideal short-circuit electric boundary conditions[7] and, using Eq.(5), to map the corresponding P_z value to E_z.

Going beyond mean field expectations and based on our numerical atomistic results, external field magnitudes corresponding to the position of transition line between phases I and II as well as phases III and IV can be obtained from universal geometrical considerations. For this we note that the volume fraction of the minority phase can be estimated from P_z as

$$c = \frac{1 - P_z\sqrt{-2\gamma'/\kappa_{\vec{q_s}}}}{2} \tag{8}$$

The transition from phase I to phase II corresponds to the percolation of the minority phase domains (see Fig. S13). Therefore, the corresponding transition field magnitude can be estimated by setting c in Eq. (8) to the percolation threshold, solving the resulting equation with respect to P_z, and then finding the critical field from the monotonic mapping between P_z and E_z (Eq. (5)) established here. The transition line between phases III and IV can be similarly estimated using the same procedure, but with c equal to the volume fraction of the minority phase dipoles corresponding to a hexagonal bubble skyrmion lattice, $c = 4\pi R^2/(3\sqrt{3}\lambda^2)$, where R denotes the equilibrium bubble radius and λ corresponds to the average distance between individual bubble domains. In a first approximation, λ can be taken equal to the ground state period of parallel stripes while R corresponds to half the width of a single stripe domain.

In figure S4, we provide the computed electric-field hysteresis of the normalized switched area S, defined as the ratio of the domain area switched by the external electric field to the total switchable area at zero field. It can be seen that S exhibits a hysteresis loop, whereby the decrease of S upon transition from monodomain to skyrmion lattice state is in delay with respect to increasing field hysteresis branch. To obtain this result, we have performed 150 simulations wherein the field was first gradually increased from 0 V/m to 60×10^7 V/m and then gradually decreased back to 0 V/m (steps of 1×10^7 V/m were used in both cases). For each simulation, the starting labyrinthine configuration was obtained with a distinct random seed through quenching the system under zero field from 650 K down to 10 K. The obtained 50 hysteresis loops were then averaged yielding the data points shown in figure S4. The normalized switched area shown therein is used to estimate the tunneling conductance of the film, via a simple circuit model described in Ref.[8]. Within this model, the domain pattern is represented by an equivalent electric circuit of capacitors connected in parallel, with each capacitor corresponding to a single [001] ("up") or [00$\bar{1}$] ("down") oriented domain. The tunneling conductance of such single-domain-capacitors depends on the polarization of the domain due to the modification of the shape of the tunneling barriers by depolarizing field (see, e.g., Ref.[9]). Owing to the parallel circuit connection, the tunneling conductance per unit area of a given domain pattern can be readily estimated as $G = G_+S_+ + G_-(1-S_+)$, where S_+ denotes the total area of "up" domains normalized by the supercell surface area, while G_+ and G_- denote the conductances of up and down domains, respectively. For instance, the conductance G_0 of the labyrinthine pattern obtained from the temperature quench at zero applied field is equal to $(G_+ + G_-)/2$ since in this case the up and down domains cover equal areas (i.e., $S_+ = 0.5$). Choosing G_0 as a reference conductance value and expressing S through a normalized reversed area $S = 2(S_+ - 0.5)$ one further obtains:

$$G = G_+S_+ + G_-(1 - S_+) = G_+\frac{1+S}{2} + G_+\frac{1-S}{2} = G_0\,(1-S) + G_+S.$$

Finally, normalizing the conductance to its value under zero applied field allows to further simplify this expression so as to leave a single numerical parameter, G_+/G_0, the value of which can be estimated in Thomas-Fermi approximation[10]. The final expression that was used to obtain the tunneling conductance hysteresis shown in Fig. 4j of the manuscript reads $G/G_0 = (1-S) + G_+/G_0S$, where G_+/G_0 was set to 0.1 in accordance with Ref. [15] and the calculated average local polarization magnitude for the zero-field labyrinthine state of 52 $\mu C/cm^2$.

In figure S5 we show the transitional process from labyrinth domain pattern to bubble skyrmions upon application of an external electric field at room temperature. Panels a, b, and c, correspond to piezoresponse force microscopy

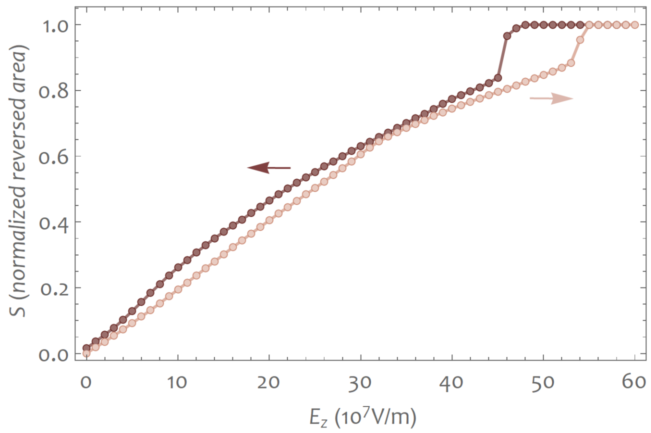

FIG. S4: Electric-field hysteresis of the normalized switched area. Fifty simulations wherein the field was first gradually increased from 0 V/m to 60×10^7 V/m and then gradually decreased back to 0 V/m were performed (steps of 1×10^7 V/m were used in both cases). For each simulation, the starting labyrinthine configuration was obtained with a distinct random seed through quenching the system under zero field from 650 K down to 10 K. The obtained 50 hysteresis loops were then averaged yielding the data points.

(PFM) amplitude images of the labyrinth domain pattern as obtained after annealing the as-grown PZT/STO/PZT system at 525 K for 10 min in air, and cooling it down to room temperature at a cooling rate of 10 to 15 K/min, an intermediate disconnected domain pattern as obtained after scanning under an AC bias of $\leq$ 300 mV via a scanning probe microscopy (SPM) probe, and the bubble skyrmions as obtained after scanning under an AC bias of $\geq$ 500 mV via a SPM probe, respectively.

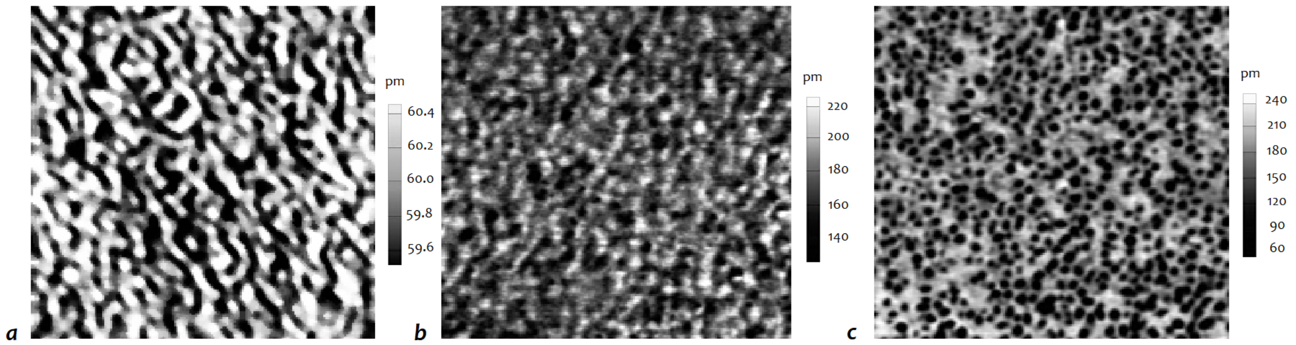

FIG. S5: PFM amplitude images of domain transitions from labyrinth towards bubble domains under electric field. (a) Labyrinth domains are obtained after annealing the as-grown PZT/STO/PZT thin films at 525 K for 10 min followed by cooling down to room temperature at cooling rate of 10-15 K/min; (b) labyrinth domain structure starts to disconnect after scanning under an AC bias of $\leq$ 300 mV via a SPM probe; (c) nanoscale bubble skyrmions are obtained after scanning under an AC bias of $\geq$ 500 mV via a SPM probe. The scan size of each of the three images is of 1μm×1μm.

In panels (a) and (b) figure S6, we provide PFM phase images corresponding to Fig. 2c1 and Fig. 2c2, respectively. Panels (c), (d), and (e) show the PFM amplitude images corresponding to Fig. 3j, Fig. 3k, and Fig. 3l, respectively. Panel (f) shows the PFM amplitude images corresponding to Fig. 4g.

The table S1 shows a comparison between numerical and experimental parameters used in this study. The differences are imputable to numerical and experimental constraints. For instance, state-of-the-art simulations do not allow for quench rates slower than Kelvin/picosecond while experimentally, such abrupt cooling is hardly achievable. Also, in contrast with simulations, the electric field profile of the SPM tip is inhomogeneous. Practically, the application of a homogenous electric field across the thin film is very challenging without a top electrode. However, introducing such a top electrode can change the screening conditions, and more importantly, make it more difficult to visualize the domain structures underneath it. To reduce the inhomogeneity, we have continuously applied the electric field on the scanned surface rather than on individual points, thereby making the average electric field equivalent at each point of the scanning area. One can also note that the values of the external electric field that were used in experiments and simulations are of the same order of magnitude. However, direct comparison of field values is challenging given that

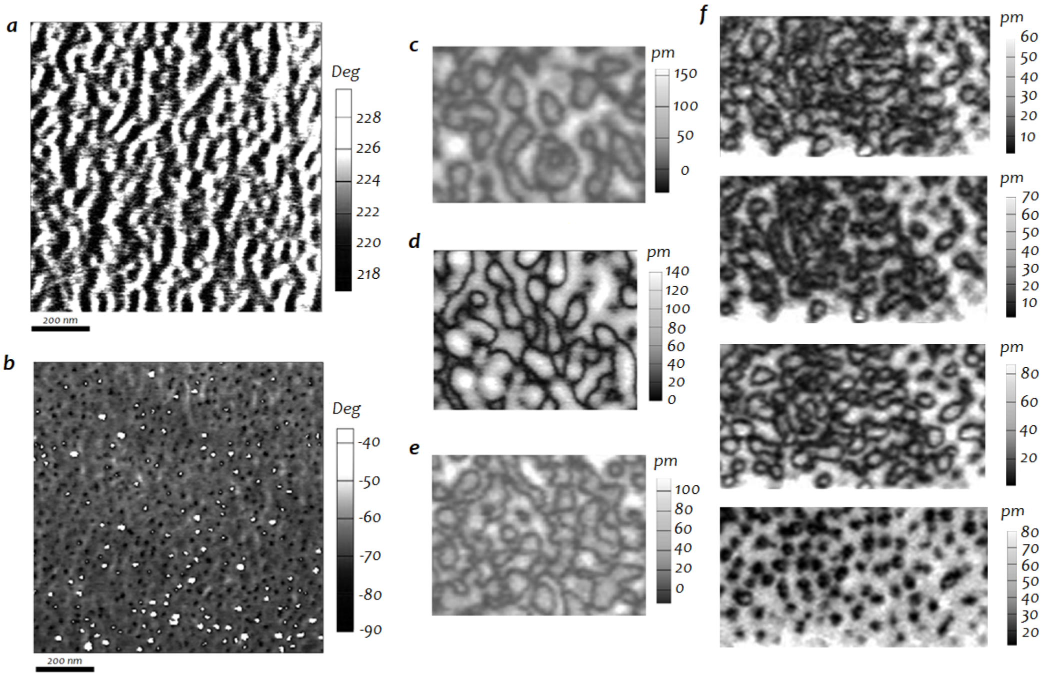

FIG. S6: Panels (a) and (b) show the PFM phase images corresponding to Fig. 2c1 and Fig. 2c2, respectively. Panels (c), (d), and (e) show the PFM amplitude images corresponding to Fig. 3j, Fig. 3k, and Fig. 3l, respectively. Panel (f) shows the PFM amplitude images corresponding to Fig. 4g.

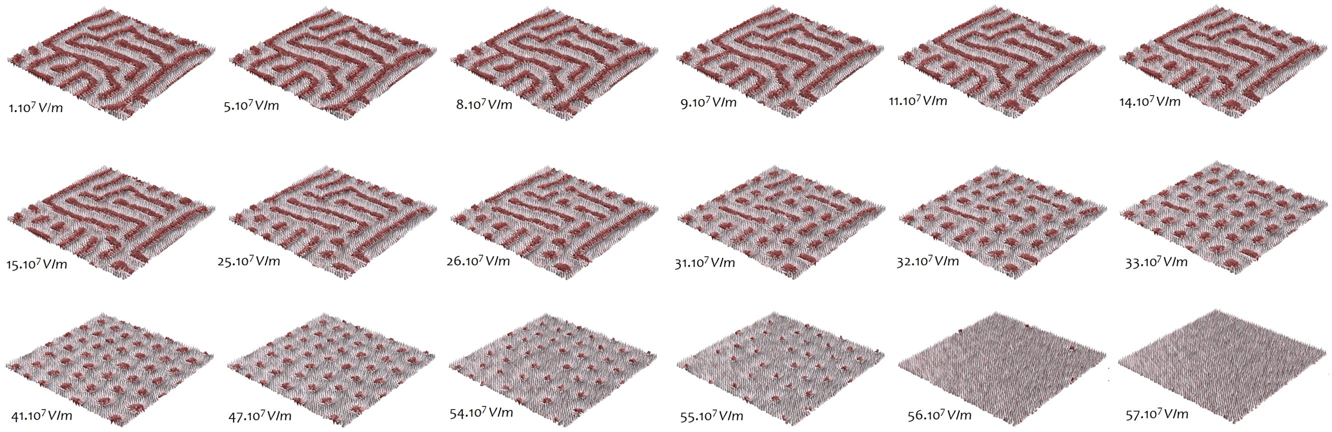

FIG. S7: Evolution with increasing electric field of the labyrinthine pattern (as obtained following a quench from 650 K down to 10 K within a 56×56×5 supercell).

multiple factors can affect the field experienced by the scanned area. Indeed, in addition to AC and DC voltages, the built-in field, the probe scan rate and the size of the scanning area intricately alter the local field. For example, a smaller area, or a slow scan rate can make the required minimum bias lower, and a higher (lower) bias could be used to accelerate (decelerate) the domain transformation, due to the change of the voltage application time per area. Furthermore, the geometry of the system that was experimentally investigated is similar but not identical to the one considered in effective Hamiltonian simulations, which can also impact the characteristic field values. Despite these quantitative disparities in the parameters summarized in the table above, we find that the qualitative similarities discussed in this study take root in equivalent physics.

In figure S7, we show the progressive transformation of the labyrinthine domain pattern (derived from the quenching procedure at zero field and down to 10 K) upon increasing the external electric field at 10 K. Note that the

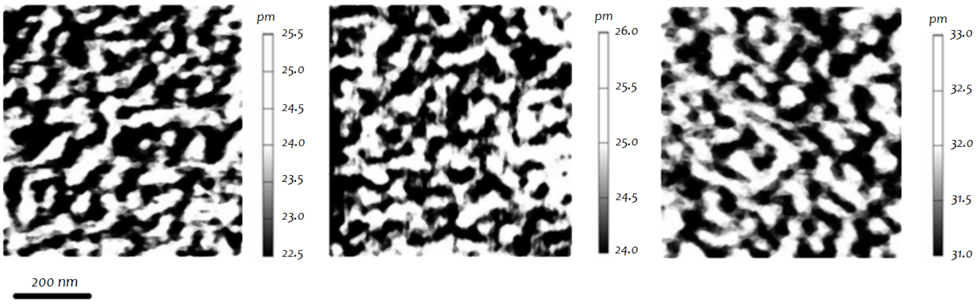

FIG. S8: PFM amplitude images of annealing derived labyrinthine domains in different areas of the sample indicating the global isotropy of the labyrinthine domain pattern.

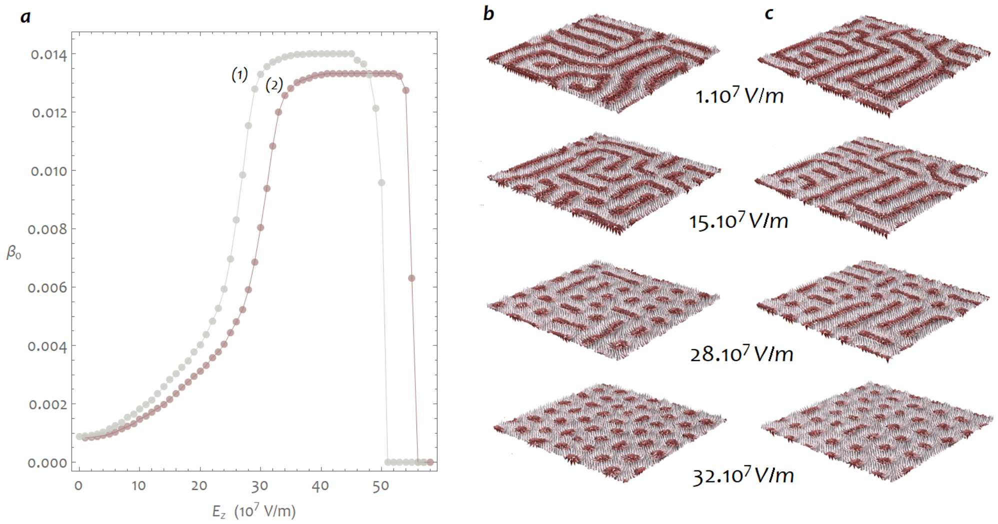

FIG. S9: Panel a corresponds to the dependence of the density of the average zeroth Betti number β_0 (density of domains) on the external electric field magnitude at 10 K, as obtained for the cases of temperature-quench under external field (blue data points, curve (1)) and progressive increase of electric field on the labyrinthine domain pattern (latter being obtained through quenching the system from 650 K down to 10 K under zero field) (red data points, curve (2)). In both cases field increments were of $1{\times}10^7$ V/m. Blue curve corresponds to an average performed, for each field value, on 200 independent (with distinct random seeds) simulations. Red curve was averaged over 200 simulations each consisting in the progressive field increase on distinct labyrinthine domain pattern. All simulations were done for a 80×80×5 supercell. Panels b and c show the dipolar configurations illustrating the dependence of the topology of the domain pattern on external electric field magnitude in both cases where the system is quenched under different fields or subjected to a progressively increasing electric field at 10 K in a 56×56×5 supercell, respectively.

corresponding simulations were performed for a 56×56×5 supercell thus yielding transition field values slightly higher than the ones reported in Fig. 2 due to finite-size effects.

One can note that the experimentally obtained labyrinthine pattern shown in Fig. 2c1 exhibits more pronounced domain roughness when compared to the numerical results shown in Fig. 2b1. We attribute these differences to the intrinsic inhomogeneities of the PZT sample such as slight thickness offsets and local defects. These inhomogeneities can locally distort the cubic anisotropy of the underlying crystalline lattice and give rise to both less orthogonal domain segments and junctions as well as stretched-like domain features. Upon collecting statistics by imaging several different areas of the sample, we note that the labyrinthine domain patterns can locally exhibit preferential direction, but remain isotropic on larger scales (see figure S8). Moreover, one can notice that numerically accessible earlier stages of the spinodal decomposition (see panel a5 of Figure 1) yield domains with a locally meandering morphology analogous to the experimental one signaled by the Referee (panel c1 of Figure 2). While the domain morphology of an ideal crystal (as in the presented simulations) can further relax and straighten, such processes are partially obstructed by the sample inhomogenities that can act as pinning centers.

The equivalency between the progressive field application at constant T and direct quenches at constant E men-

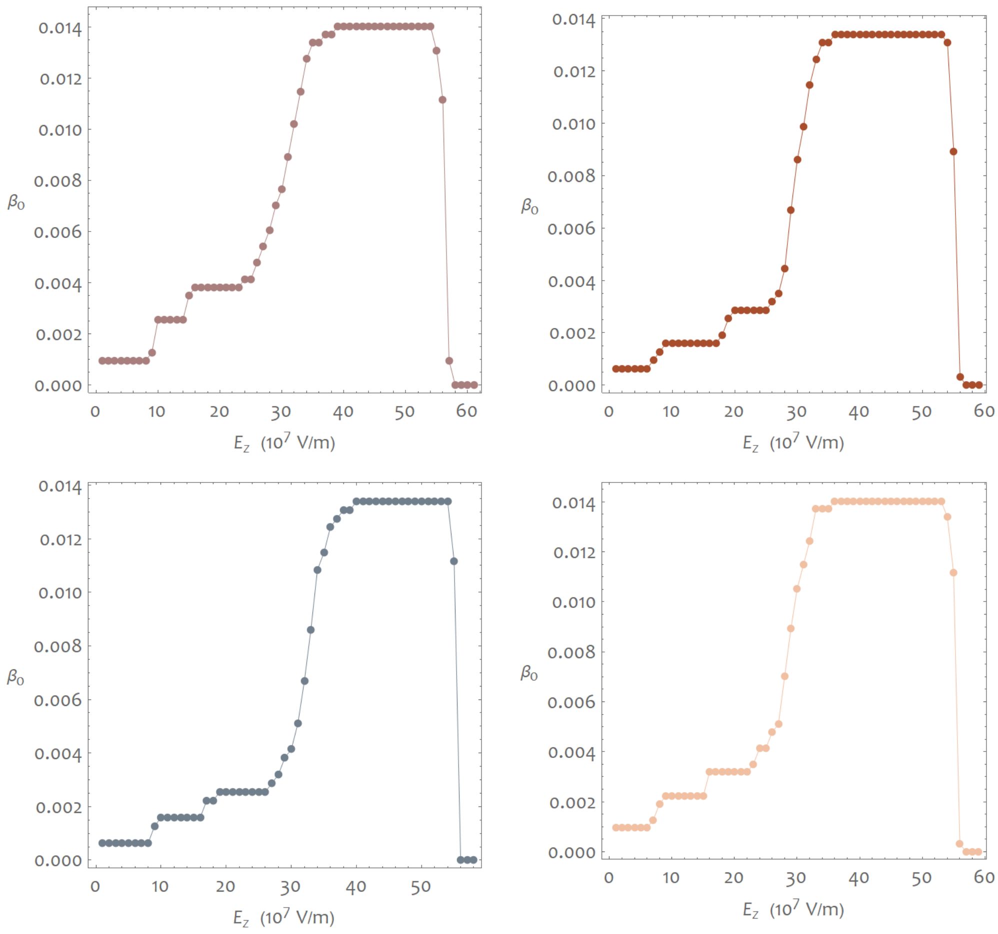

FIG. S10: Examples illustrating the dependence of the density of the zeroth Betti number β_0 (density of domains) on the external electric field magnitude at 10 K, as obtained upon progressively increasing the applied electric field magnitude. The four panels correspond to four out of 200 statistically independent simulations.

	Simulations	Experiments
Film thickness	2 nm	7 nm
Heating temperature	650 K	525 K
Quenching temperatures	10 K to 410 K by steps of 50 K	298 K
Quenching rate	1 K/ps	10-15 K/min
Time after quench	200 ps	10-60 mins
Field application	0-70 $\times$ 10^7 V/m<br>uniform DC bias	local DC: 0-25 $\times$ 10^7 V/m<br>local AC: 0-7 $\times$ 10^7 V/m
Smallest domain size	1.8 nm	<5 nm

TABLE S1: Table contrasting the main parameters of simulations and experiments.

tioned in the manuscript is supported by Fig. S9, wherein data points are obtained upon averaging over 200 distinct realizations for each of the two cases. As it can be seen, the two data sets, albeit not identical, qualitatively exhibit comparable behavior, reflecting the similarity between the topological transformations occurring in the system during one or the other field treatment (panel b of Fig. S9). The main difference between the two curves resides in the shift of characteristic field values at which such transitions onset (shift of $\sim 5\times10^7$ V/m). Given the numerical and experimental constraints (see table S1), we consider such differences between the two curves inconsequential with respect to the main conclusions of the present study.

While averaging over many distinct quenches or field-sweeps yields a smooth behavior of the average β_0 (see Fig. S9), individual realizations recurrently feature plateaux in their evolution with electric field (see Fig. S10). The smooth behavior of averaged β_0 can thus be attributed to a distribution of the field values delimiting each of the plateau-

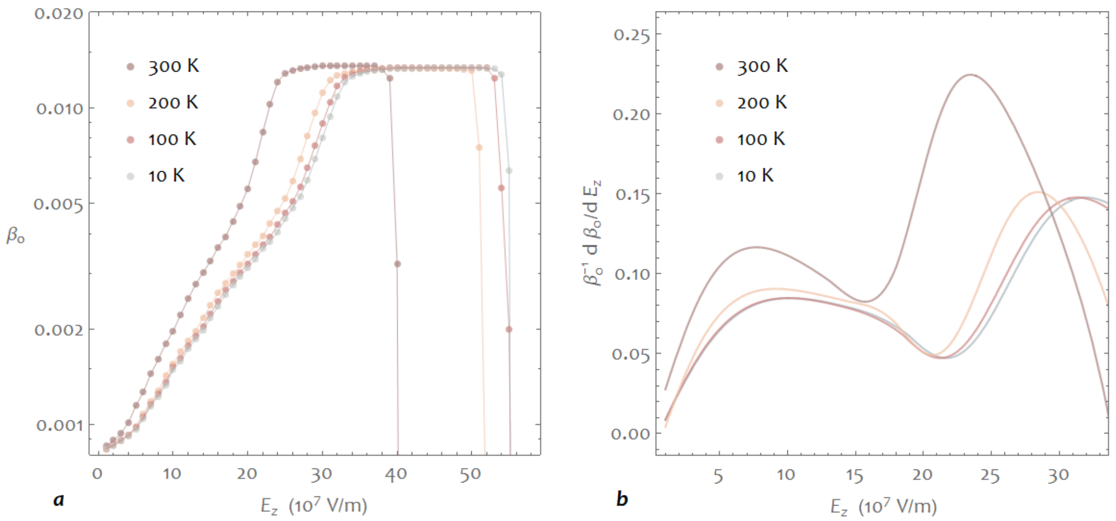

FIG. S11: Panel a provides the dependence of the density of the average zeroth Betti number β_0 on the external electric field magnitude at 10 K, 100 K, 200 K, and 300 K as obtained upon progressively increasing the electric field magnitude. For each temperature, the transition to the monodomain phase is identified with the field value at which $\beta_0 = 0$. Each curve corresponds to an average over 200 independent simulations. Panel b shows the logarithmic derivatives of the average β_0 obtained through piecewise fitting the curves displayed in panel a with polynomial functions. The extrema of these derivatives are used to identify the transitions between the different topologies characterizing phases I, II, III, and IV.

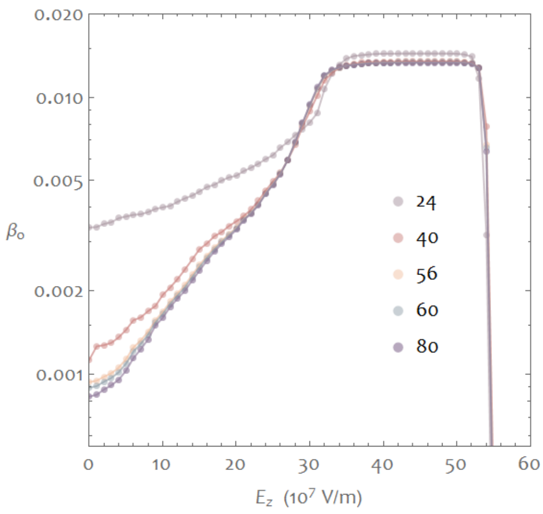

FIG. S12: Dependence of the density of the zeroth Betti number β_0 on the external electric field magnitude at 10 K for increasing supercell lateral size (24, 40, 56, 60, and 80) as obtained upon progressively increasing the electric field magnitude. Each curve corresponds to an average over 200 independent simulations.

regions, as well as from the distributions of their extent and their associated β_0 value. However, one can notice the persistence of shoulders in the averaged data (panel a of Fig. S9), and we find that different phases can be traced through the inflection points of the logarithm of the averaged curve (panel a of Fig. S11). The use of the logarithmic scale is motivated by the geometric growth of the domain number during the disconnection process. Note that the position of the inflection points is well converged (increasing the statistical ensemble of distinct realizations beyond 150 does not affect their positions). The transition lines (I to II to III to IV) are therefore associated with the extrema of the logarithmic derivative of the average β_0 at each temperature (panel b of Fig. S11). The last transition line (IV to V) is identified with the field value at which average $\beta_0 = 0$ (see panel a of Fig. S9). The resulting phase diagram is shown in panel a of Fig. 2 of the manuscript, wherein lines interpolate between the data points obtained for 10 K and from 50 K to 350 K by steps of 50 K.

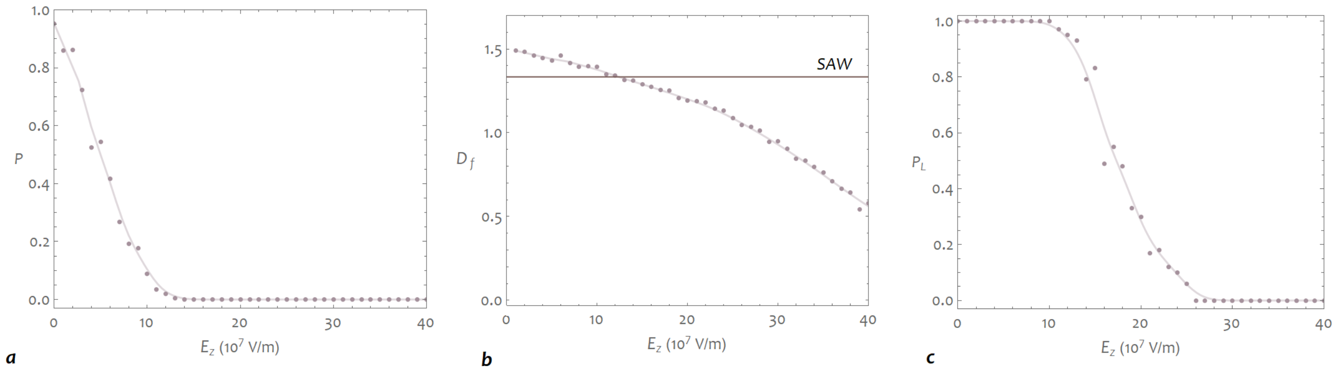

FIG. S13: Panels provide the dependence of (a) the probability of occurrence of a percolating cluster, (b) the fractal dimension of domain pattern calculated through box counting method (c) the probability of occurrence of domains featuring a change in the propagation direction, on the magnitude of the electric field at 10 K. The horizontal line in panel b corresponds to 4/3, the fractal dimension of a self-avoiding walk in two dimension (SAW). All data points were obtained by averaging the results over 200 independent temperature quenches at each value of the electric field. Lines are guides to the eye.

Collecting statistics for several lateral sizes indicates that convergence of β_0 can be considered achieved for $56\times56\times5$ supercell size (see Fig. S12). Though believed to be accurate in the thermodynamic limit (limit of infinite dimensions with infinitely weak, infinitely long-ranged forces), the exactness of a mean field approximation can remain elusive. Two-dimensional ferroelectrics (with reduced dimensionality and enhanced fluctuations when compared to their bulk form) intrinsically subjected to relatively strong and long-ranged dipole-dipole interaction often depart from mean-field expectations, despite the size increase. Indeed, as mentioned in the manuscript, the progressive transition from spinodal decomposition to nucleation contrasts with mean-field expectations of a sharp boundary separating the two (see panel b of Fig. S3), consistently with the idea that the mechanism of nucleation approaches the mechanism of spinodal decomposition continuously as the spinodal curve is approached[11]. In a sense, the departure from mean-field is encapsulated in the intermediate phases of the phase diagram, wherein phases II and III are transitional phases (in the thickness of the boundary) between phases I and IV, associated with spinodal decomposition and nucleation, respectively.

It may be worthwhile noting that alternative geometrical procedures can be instructive to identify the position of the transition lines. For instance, the identification of the transition from the connected (phase I) to the disconnected labyrinthine pattern (phase II), can be readily achieved through the examination of the probability of occurrence of a percolating cluster (percolation threshold[12]), or of the field value at which the fractal dimension of the labyrinthine pattern meets that of two-dimensional self-avoiding random walks (4/3 in two dimensions). As for the transition from the disconnected labyrinth (phase II) to the mixed meron-bimeron phase (phase III), one could identify the probability of occurrence of domains featuring a change in propagation direction (i.e., domains with “L”-like morphologies). These alternative approaches are shown in Fig. S13, wherein the obtained results were averaged over 200 independent temperature quenches at each value of the electric field.

[1] Hoshen, J. & Kopelman, R. Percolation and cluster distribution. I. Cluster multiple labeling technique and critical concentration algorithm. Phys. Rev. B 14, 3438-3445 (1976).

[2] Prokhorenko, S., Nahas, Y. & Bellaiche, L. Fluctuations and topological defects in proper ferroelectric crystals. *Phys. Rev. Lett.* **118**, 147601 (2017).

[3] Ponomareva, I., Naumov, I. I., Kornev, I., Fu, H., & Bellaiche, L. Atomistic treatment of depolarizing energy and field in ferroelectric nanostructures. *Physical Review B* **72**, 14 (2005).

[4] Chaikin P. M. & Lubensky, T. C. Principles of Condensed Matter Physics (Cambridge University Press, Cambridge, UK, 1995)

[5] Vaks, V. G. Introduction to the Microscopic Theory of Ferroelectrics, Nauka, Moscow (1973)

[6] Peskin, M. E. & Schroeder, D. V. An introduction to quantum field theory, Westview, Boulder (1995)

[7] Pertsev, N. A., Zembilgotov, A. G. & Tagantsev, A. K. Effect of Mechanical Boundary Conditions on Phase Diagrams of Epitaxial Ferroelectric Thin Films. *Phys. Rev. Lett.* **80**, 1988 (1998)

[8] Boyn, S. *et al.* Learning through ferroelectric domain dynamics in solid-state synapses. *Nature Communications* **8**, 14736 (2017).

[9] Tsymbal, E. Y. Tunneling across a ferroelectric. *Science* **313**, 181-183 (2006).

[10] Zhuravlev, M. Ye., Sabirianov, R. F., Jaswal, S. S. & Tsymbal, E. Y. Giant Electroresistance in Ferroelectric Tunnel Junctions. *Phys. Rev. Lett.* 94, 246802 (2005).
[11] Cahn, J. On spinodal decomposition. *Acta Metallurgica* **9**, 795-801 (1961).
[12] Stauffer, D. An Introduction to Percolation Theory (Taylor and Francis, London, 1985)
